# Density-Dependent Effects on Group Size Are Sex-Specific in a Gregarious Ungulate

**DOI:** 10.1371/journal.pone.0053777

**Published:** 2013-01-09

**Authors:** Eric Vander Wal, Floris M. van Beest, Ryan K. Brook

**Affiliations:** 1 Department of Animal and Poultry Science, College of Agriculture and Bioresources, University of Saskatchewan, Saskatoon, Saskatchewan, Canada; 2 Département de Biologie, Université de Sherbrooke, Sherbrooke, Québec, Canada; University of Western Ontario, Canada

## Abstract

Density dependence can have marked effects on social behaviors such as group size. We tested whether changes in population density of a large herbivore (elk, *Cervus canadensis*) affected sex-specific group size and whether the response was density- or frequency-dependent. We quantified the probability and strength of changes in group sizes and dispersion as population density changed for each sex. We used group size data from a population of elk in Manitoba, Canada, that was experimentally reduced from 1.20 to 0.67 elk/km^2^ between 2002 and 2009. Our results indicated that functional responses of group size to population density are sex-specific. Females showed a positive density-dependent response in group size at population densities ≥0.70 elk/km^2^ and we found evidence for a minimum group size at population density ≤0.70 elk/km^2^. Changes in male group size were also density-dependent; however, the strength of the relationship was lower than for females. Density dependence in male group size was predominantly a result of fusion of solitary males into larger groups, rather than fusion among existing groups. Our study revealed that density affects group size of a large herbivore differently between males and females, which has important implications for the benefits e.g., alleviating predation risk, and costs of social behaviors e.g., competition for resources and mates, and intra-specific pathogen transmission.

## Introduction

Density-dependent processes are fundamental to population ecology [1], [2], which have important implications for group size dynamics of social species [3]–[9]. Fitness returns from social behaviors, such as group size, exist as trade-offs between costs and benefits (e.g., minimizing predation risk [10] or social foraging [11]). Changes in group size as a function of population density (i.e., competition) have been reported for several social species (see [12] for a review); however, sex-specific density effects have rarely been considered, although sexual segregation is common among social ungulates [13]. Moreover, the mechanisms driving such relationships (i.e., density or frequency dependence) remain untested.

Social behaviors have important costs and how conspecific density affects these costs is affected by competition [14]–[17] and pathogen transmission [12], [18], [19]. Whether a social behavior, such as group size, varies with density and whether these changes occur in a non-linear (i.e., density and negative density)- vs. linear (i.e., frequency)-dependent fashion could reveal how such costs may affect individuals and populations. For example, if intra-specific interaction rates are affected by group size, a density-dependent response of group size indicates that the costs of social behaviors will not only be greater at high density, but are likely to be exponentially so. Furthermore, a non-linear response suggests that thresholds exist below which certain costs (e.g., disease persistence [20]) may no longer be germane.

Here we tested whether individual exposure to group size changed with population density and whether those changes were density- or frequency-dependent using a population of elk (*Cervus canadensis*) that fluctuated between1.20 to 0.67 elk/km^2^ during an experimental reduction from 2002 to 2009. Due to spatial and social sexual segregation, costs of sociality may differ between male and female elk [21]–[23]; for example, injuries in males either to formative antlers, or to mature antlers [21], [24]. Conversely, females may benefit from increased group size. For example, group vigilance is predicted to offset costs of vigilance for females feeding with young-at-heel [25]. We expected that at any given density male group size would be smaller than female group size (prediction 1). We also expected that group size would increase with density (e.g., as in chamois, *Rupicapra pyrenaica* [6]) in a density-dependent fashion for both sexes (prediction 2); although we have no evidence to suggest this will occur indefinitely for both sexes (see [26] for density effects on dyadic interactions, and [27]). Describing general mechanisms of change in group size related to density is critical, however, it does not reveal among which groups these changes occur. Here we assume a random Brownian model of animal movements within a finite area [28]; decreased spacing among individuals with increased density [29]; and the regular intergroup fusions known to occur among elk [30]. Given these constraints we expected to observe either more large groups with no change in dispersion (prediction 3a, [6]) or more small but less dispersed groups (prediction 3b, [4]) as population density increased.

## Methods

### 1. Animal Ethics Statement

This work was approved and performed in accordance with the Canadian Council on Animal Care. It was governed by two separate animal care protocols: University of Manitoba #F01-037 (2002–2005) and University of Saskatchewan, #20060067 (2006–2009). Furthermore it was conducted in accordance with a Parks Canada Environmental Assessment and Research Permit.

### 2. Study area

Our study area included Riding Mountain National Park (RMNP, 3,000 km^2^; 50°51′50″N 100°02′10″W) and is located in Manitoba, central Canada. RMNP falls within the Prairie Parkland and Boreal Plains transition zone [31]. Elk (*Cervus canadensis manitobensis*) live primarily within and near the periphery of the preserve ( Figure S1, Supplementary Material) and are regularly depredated upon by wolves (*Canis lupus*; [32]), which have remained stable at about 100 animals during our study (Parks Canada, unpublished data). Forest cover consists of aspen (*Populus tremuloides*) mixed with conifer (e.g., *Picea glauca* and *Pinus banksiana*), interspersed with marshlands. As wildfire is infrequent and timber harvest is prohibited within the national park, these forests changed little during the course of our study apart from the decadal scale of natural forest succession [33]. The regional elk population, however, fluctuated dramatically during this study (Parks Canada unpublished data). The elk population has been actively managed [34], predominantly through the number of licenses available to hunters around RMNP. During the course of this study the elk population was being experimentally reduced as an attempt to reduce the economic impacts of elk in the region. These include agricultural damage by elk and the risk of bovine tuberculosis (*Mycobacterium bovis*) present in the elk population being transmitted to cattle [35], [36]. As such, the regional elk population density was actively decreased from a high of 3600 to a low of 2000 through a federal and provincial government joint management program, which primarily involved longer hunting seasons and increased number of hunting tags available for elk in the hunting zones immediately adjacent to Riding Mountain National Park elk population.

### 3. Group size estimates

Elk (*n* = 178 F, 135 M) were equipped with Very High Frequency radio-collars from 2002–2005, 2007–2009. We located each animal during daylight hours (0800–1900 hr) 1–16 times every fortnight by aircraft using standard methods [37]. During telemetry flights we collected geo-referenced visual observations of collared individuals and counted the number of neighboring conspecifics (i.e., exposure to group size, *sensu* [12]). Exposure to group size (hereafter group size) is the number of conspecifics to which a focal individual is exposed. As such we did not measure group composition. Yearlings were counted in the totals for group size; however, young of the year were not included in counts of group size. Group size equaled the number of elk proximal to the collared individual (*i* in group *A*); where the estimated distance from the animal *i* to any individual (*j*) in *A* was less than the distance between *j* in group *A* and an individual (*k*) in a potentially separate group, *B*. It was very rare to observe groups without obvious discontinuous breaks in their distribution.

### 4. Population density

Changes in population size determine changes in large-scale population density (i.e., elk abundance/available habitat) because RMNP is an insular system [38], [39] with elk associated closely to the park [40] and little native habitat outside of the park [41]. Furthermore, the entire RMNP is available to and is used by elk, as verified by >30 years of aerial surveys and telemetry work (Parks Canada, unpublished data). Population density estimates were derived from 25% coverage annual winter aerial surveys conducted by Parks Canada staff in RMNP. The method is described by Rounds 1981 [42] and now includes *n* = 68 transects across the park (Figure S1, Supplementary Material). Transects were 200 m wide and conducted annually in January at an altitude of 120 m at 120 km/hr by the same two trained observers every year in a fixed-wing aircraft (Figure S1). Transects ranged from 8.5–24.0 km^2^ due to the shape of RMNP, totaling 745 km^2^. During the study period identical transects were flown annually using the same pilot and observers to ensure a consistent estimate of population size across years. Thus, we assumed there was little variance in precision of population estimates and as such differences between years remain biologically relevant (see Text S1, Supplementary Material for details).

### 5. Density- vs. frequency-dependent response to mean group size

Following Pepin and Gerard [6] we first tested for a relationship between mean group size and population density. Sex-specific mean group sizes were calculated for each observed annual population density (0.67, 0.67, 0.76, 0.76, 0.86, 1.20 elk/km^2^; *n* = 258, 460, 85, 545, 1264, 758, respectively). We used an information-theoretic framework to test whether change in mean group size would be frequency-dependent (i.e., linear) or density-dependent (i.e., curvilinear) by comparing linear and quadratic models, using inverse variance weighted general linear models (prediction 1 and 2).

### 6. Density- vs. frequency-dependent response of unadjusted group size controlling for seasonal sight-ability

We tested whether individual response of unadjusted (i.e., raw) group size to density concurred with predictions from changes in mean group size. Here we divided the group size observations into two seasons to additionally account for any possible affects that may follow from intra-annual changes in sight-ability due to canopy cover: deciduous canopy present, April – September (n = 997); and deciduous canopy absent, October – March (n = 2,373) following from Vander Wal et al. [43]. Again, we used an information-theoretic framework to test whether change in group size would be similar to frequency-dependent or density-dependent by comparing linear and quadratic models with season as a fixed factor. However, because count data approximate a Poisson distribution we used general linear models with Poisson distribution (prediction 1 and 2), thus we were unable to fully linearize a test of frequency dependence.

### 7. Changes in frequency of group sizes encountered

We binned sex-specific group sizes following Hebblewhite and Pletcher [9] using five biologically meaningful group sizes: 1, 2–5, 6–12, 12–30, >30. To test how the distribution of different sized groups would change with population density (prediction 3a), we calculated the probability of the observer encountering an elk group of bin size *x* during each sample day. The results of each sample day were divided into proportions of observations of each bin. We used generalized linear models for proportion data with an over-dispersed binomially distribution to quantify the relationship between probability of encountering a group of a given size with changes in density.

### 8. Changes in group dispersion (binned by size)

Either in addition to or in lieu of changes in group size, group dispersion can also increase with increased population density (prediction 3b). To test how the dispersion of groups changes with density we calculated the mean nearest neighbor distance between sex-specific groups on a given sample day. First we used a general linear mixed model to test whether mean nearest neighbor distance differed between sexes. Mean nearest neighbor distance was log transformed to improve normality of regression residuals [44]; density and bin size were added as random intercepts to control for changes in population size and so that mean nearest neighbor distance may vary across differently sized bins.

Subsequently we tested whether dispersion changed with population density for differently sized groups (i.e., each bin). Individual general linear models were used to regress log(mean nearest neighbor distance) against density for each unique bin. All analyses were performed in R (version 2.13; [45]).

## Results

### 1. Density- vs. frequency-dependent response to group size

**Figure 1:**
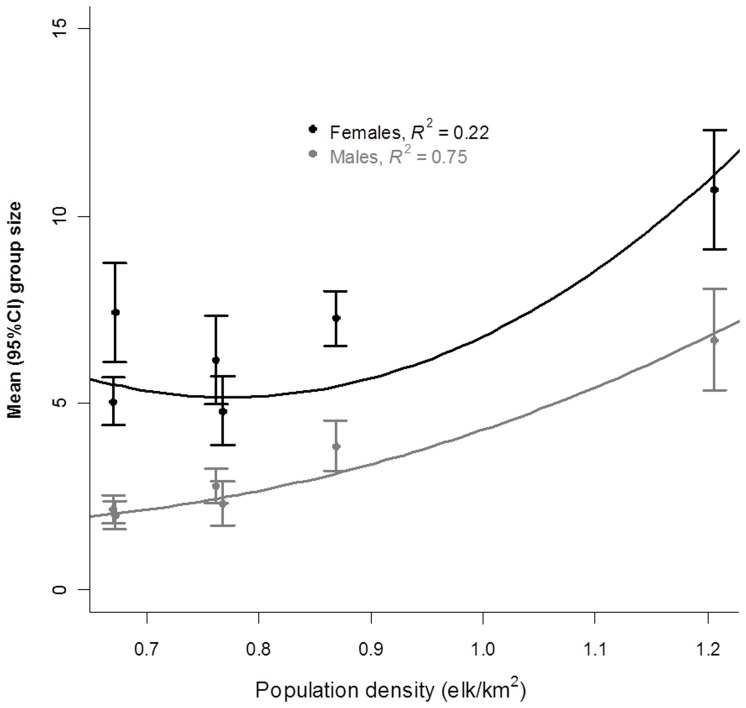
Relation between mean group size and population density for female (black) and male (gray) elk in Riding Mountain National Park (2002–2005 and 2007–2009). Lines are quadratic fits to the mean group size data illustrating: (a) density-dependent change in females, including negative density-dependence at low density; and (b) weak density dependence for males.

**Figure 2:**
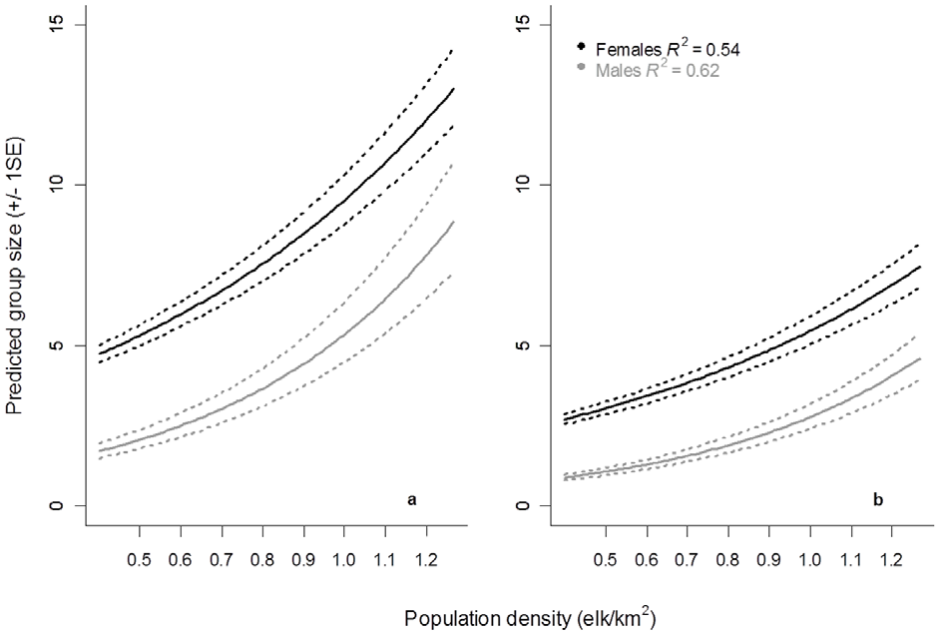
Predicted change in unadjusted group size for female and male elk in Riding Mountain National Park (2002–2005 and 2007–2009); here the dichotomy between density-dependent response in group size for females and males is pronounced. These models also control for seasonal sight-ability bias due to canopy cover (a) October – March, (b) April – September.

Changes in mean group size and raw group size corrected for seasonal sight-ability were sex-specific. At each density females were observed in larger groups than males, supporting prediction 1 (Figure 1). Unequivocally mean group size increased with density as expected (Table 1, prediction 2); however for females this relationship relies on observations taken at the highest density (Text S2, Supplementary Material, Tables S1 and S2). The sex-specific dichotomy in response to density did not change with season (Figure 2 and Table 2).

**Table 1:** Comparison of frequency – (*FD*), and density – (*DD*) response of mean group size 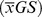 for female (*_F_*) and (*_M_*) elk to population size (*D_N_*) in Riding Mountain National Park over six years (2002–2004, 2007–2009) during an intentional population reduction.

	*A priori* Model		Coefficients and *P*-value		*R* ^2^	ΔAIC	AIC*_w_*
Females	*FD*	*χGS_F = _β*_1_ *(D_N_)+β*_0_	*β*_1_ = 3.1×10^−3^	*P* = 0.37	0.00	3.88	0.41
	*DD*	*χGS_F = _β*_1_ *(D_N_)+β*_2_ *(D_N_)* ^2^ *+β*_0_	*β*_1_ = −1.6×10^−2^	*P* = 0.34	0.22	0	0.59
			*β*_2_ = 3.54×10^−6^	*P* = 0.28			
Males	*FD*	*χGS_M = _β*_1_ *(D_N_)+β*_0_	*β*_1_ = 6.8×10^−4^	*P* = 0.02	0.71	1.25	0.34
	*DD*	*χGS_M = _β*_1_ *(D_N_)+β*_2_ *(D_N_)* ^2^ *+β*_0_	*β*_1_ = −3.6×10^−4^	*P* = 0.42	0.75	0	0.65
			*β*_2_ = 1.2×10^−6^	*P* = 0.23			

**Table 2:** Comparison of density – (*DD*), and negative density–dependent (*NDD*) response to unadjusted group size 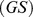 for female (*_F_*) and (*_M_*) elk to population size (*D_N_*) by season (*S_1,2_*)^1^ in Riding Mountain National Park over six years (2002–2004, 2007–2009) during an intentional population reduction.

	*A priori* Model		Coefficients and *P*-value		*R* ^2^	ΔAIC	AIC*_w_*
Females	*DD*	*GS_F = _β*_1_ *(D_N_)+(S*_1,2_ *)*, log link function	*β*_1_ = 3.9×10^−4^	*P*<0.001	0.54	0	0.70
			S_1_^ = 0.53^	*P*<0.001			
			S_2_ = 0.55	*P*<0.001			
	*NDD*	*GS_F = _β*_1_ *(D_N_)+β(D_N_)* ^2^ *+(S*_1,2_ *)*, log link function	*β*_1_ = 7.4×10^−5^	*P*<0.001	0.54	1.7	0.30
			*β*_2_ = −6.0×10^−8^	*P* = 0.05			
			S_1_ = 5.3×10^−2^	*P* = 0.84			
			S_2_ = 5.6×10^−1^	*P*<0.001			
Males	*DD*	*GS_M = _β*_1_ *(D_N_)+(S*_1,2_ *)*, log link function	*β*_1_ = 6.3×10^−4^	*P*<0.001	0.61	0	0.99
			S_1_ = −8.6×10^−1^	*P*<0.001			
			S_2_ = 6.5×10^−1^	*P*<0.001			
	*NDD*	*GS_M = _β*_1_ *(D_N_)+β(D_N_)* ^2^ *+(S*_1,2_ *)*, log link function	*β*_1_ = 2.3×10^−3^	*P*<0.001	0.62	18.0	0.01
			*β*_2_ = −2.8×10^−7^	*P*<0.001			
			S_1_ = −3.2×10^−1^	*P*<0.001			
			S_2_ = 6.5×10^−1^	*P*<0.001			

1 Season is divided into two periods of unequal sight-ability: April – September (1) with deciduous canopy cover present; and October – March (2) in the absence of deciduous canopy cover, see [43] for details.

For females, mean group size changed in a density-dependent fashion with negative density dependence occurring at population densities ≤0.70 elk/km^2^ (Table 1 and Figure 1). The density-dependent model for females explained 22% of the variation present in the data (Table 1), whereas the frequency-dependent model explained effectively no variance. Density-dependence was corroborated for raw group size adjusted for sight-ability bias (*R* ^2^ = 0.54, Table 2). However, we were unable to distinguish between an exponential model of density dependence and a negative density-dependent model (Δ*AIC <2*, Table 2 and Figure 2).

For mean male group size we were unable to differentiate between frequency- and density-dependent changes in mean group size (Table 1). The models with the highest *AIC_w_* were typically density-dependent (curvilinear; Figure 1 and Tables 1). However, the curve indicated that this density dependence is nearly linear (Figure 1). There was strong evidence against negative density-dependence for raw group size in males; strongly favoring the exponential model of density-dependence (*AIC_w_* exponential = 0.99 vs. negative density-dependent = 0.01, Table 2).

### 2. Changes in frequency of group sizes encountered

**Figure 3:**
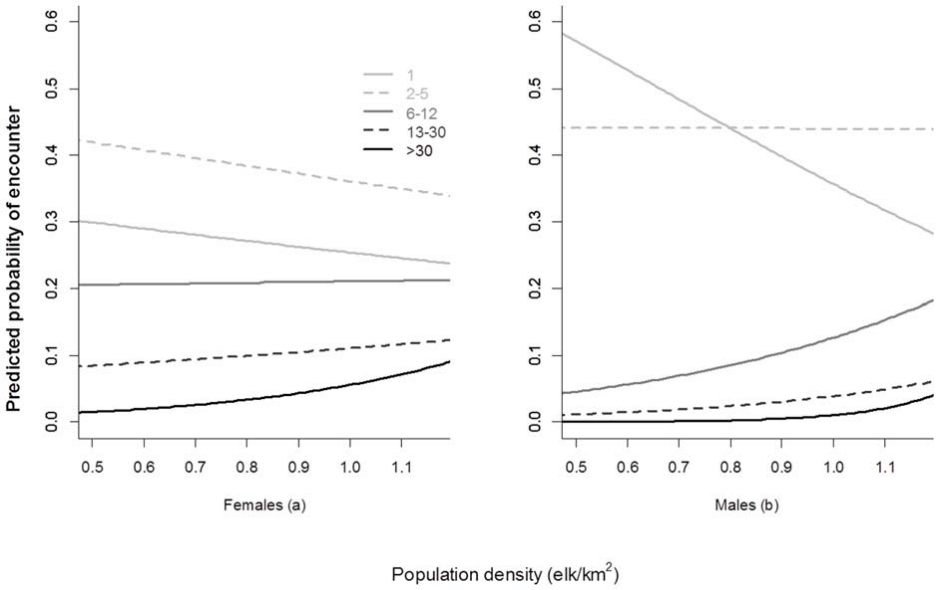
Predicted probability of encounter rate of groups (binned by size) for female (a) and male (b) elk with changes in population density in Riding Mountain National Park (2002–2005 and 2007–2009).

As population density increased, the probability of encountering larger groups increased, supporting prediction 3a (Table 3). For females the probability of observing groups of >14 individuals increased with population density (Figure 3a), while the probability of observing groups of 6–12 individuals remained similar and decreased for groups of 1 or 2–5 individuals. The probability of observing a solitary male declined dramatically with decreasing population density, whereas the probability of group size of 2–5 individuals increased only marginally with increasing population density (Figure 3b). Conversely, as population density increased, the probability of observing groups of 6–12 males also increased.

**Table 3:** Regression of daily probability of encounter with groups (binned by size) as a function of population size for elk in Riding Mountain National Park (2002–2004, 2007–2009).

	Bin Size	Direction	Coefficient	*SE*	*P*-value
Females	1	−	−1.51×10^−4^	5.02×10^−5^	**<0.001**
	2–5	−	−1.64×10^−4^	3.65×10^−5^	**<0.001**
	6–12	+	2.11×10^−5^	4.32×10^−5^	0.68
	13–30	+	2.00×10^−4^	6.33×10^−5^	**0.002**
	>30	+	8.89×10^−4^	8.56×10^−5^	**<0.001**
Males	1	−	−5.84×10^−4^	5.56×10^−5^	**<0.001**
	2–5	−	4.18×10^−6^	5.79×10^−5^	0.99
	6–12	+	7.36×10^−4^	7.16×10^−5^	**<0.001**
	13–30	+	8.00×10^−4^	1.10×10^−4^	**<0.001**
	>30	+	2.36×10^−3^	2.42×10^−4^	**<0.001**

### 3. Changes in group dispersion (binned by size)

**Figure 4:**
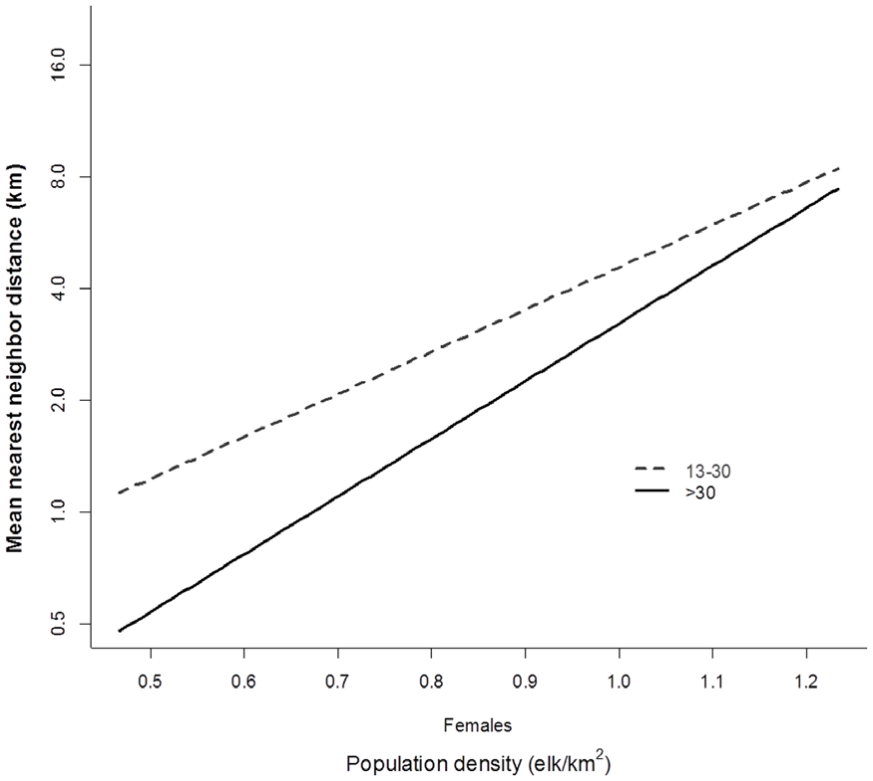
Predicted changes in group dispersion (binned by size) for female elk with changes in population density in Riding Mountain National Park (2002–2005 and 2007–2009).

Female groups were less dispersed than male groups (*P* <0.001). However, contrary to prediction 3b, large female groups became more dispersed as population size increased (Table 4, Figure 4), although results were only significant for groups >13 individuals. We found no indication that male groups became more or less dispersed as density increased as all results were non-significant (Table 4).

**Table 4:** Regression of group dispersion (binned by size) as a function of population size for elk in Riding Mountain National Park (2002–2004, 2007–2009).

	Bin Size	Direction	Coefficient	*SE*	*P*-value (*R^2^*)
Females	1	+	9.99×10^−5^	1.74×10^−4^	0.56 (–)
	2–5	+	1.04×10^−4^	1.34×10^−4^	0.43 (–)
	6–12	+	4.18×10^−4^	2.32×10^−4^	0.07 (0.02)
	13–30	+	8.74×10^−4^	4.00×10^−4^	**0.03** (0.08)
	>30	+	1.11×10^−3^	3.81×10^−4^	**<0.01** (0.30)
Males	1	−	−1.11×10^−4^	1.48×10^−4^	0.43 (–)
	2–5	+	2.06×10^−4^	1.38×10^−4^	0.14 (–)
	6–12	−	−1.93×10^−4^	4.56×10^−4^	0.67 (–)
	13–30	−	−6.52×10^−4^	1.32×10^−4^	0.63 (–)
	>30	_	__	__	__

## Discussion

Although many studies have investigated the effect of predation on group size (e.g., [9], [25]), particularly in elk, the effect of population density is often discounted (but see [4]). We tested for changes in mean, individual unadjusted, and binned, sex-specific group sizes as a function of population density. We focused on estimates of group size distributed throughout the population and large scale patterns in population density (size) during an intentional population reduction. Changes in group size were density-dependent irrespective of sex. However, we present evidence for negative density dependence in female groups' size and that density dependence in male group size was weak. Furthermore, we illustrated that female groups became larger (>13 individuals) and less dispersed as population size increased. As such, females were more likely to aggregate with increased population density. Similarly, as population density increased, observing solitary males became uncommon, which increased observations of male groups of >6 individuals in size. However, there was no evidence that the distribution of male groups changed with density.

Our study provides significant evidence that mean group size does increase with population density. The density-group size relationship was clearly sex-specific (as expected by prediction 1). These findings contrast with an earlier study by Profitt et al. [4], which found that groups increased in size in a linear fashion. Groups were not assigned to be predominantly male or female in Proffitt et al. [4], and estimates of group size were not obtained from focal individual observations. At any given density we clearly demonstrated that females formed larger groups than males. Our models also indicated that females predominantly followed a density-dependent response to population size. The response was curvilinear (partial support for prediction 2). Males' response was equivocal when considering mean group size. However, when accounting for sight-ability and unadjusted group size estimates we found evidence for weak density-dependent changes in group size as a function of population density. We argue, therefore, that the response of males to density is also weakly density-dependent. Typically our best models explained considerably more variation in changes in mean or unadjusted-group sizes than previously demonstrated (e.g., [46], *R^2^* = 0.07 vs. Table 1 and 2). This is likely due to our focal individual sampling, decomposition into predominantly sex-specific groups, and in some cases non-linear response to increasing density.

Johnson [27] suggested that sex-based difference in group size in response to density is related to breeding strategy and intra-sexual competition. For kangaroos [27] and chamois [6] groups size was observed saturating in a logarithmic fashion suggesting that group size will increase until an allegedly optimal size is reached [12]. Sibley [47], however, suggested that realized group size should be marginally larger than optimal. Ultimately optimal group size can only be evaluated with cost:benefit data (i.e., fitness). However, we observed no indication that group size reached an upper or allegedly optimal size at the highest observed density in RMNP (as predicted in [12]). Rather than an upper limit to group sizes, there appears to be a minimum group size for female at approximately 5 individuals. The same minimum group size did not occur for males. However, the existence of lone males was very sensitive to changes in density. This indicates that males are more likely than females to be solitary at low population density. Our data corroborate that intra-sexual competition may be higher in males than in females, as is evidenced by smaller groups at low densities and a slower increase in group size with increasing density than for females.

Decomposing groups into biologically relevant bins revealed clear patterns. Indeed, Proffitt et al. [4] described that, independent of sex, increases in group size with population density did occur among the largest groups (99^th^ percentile). We extend the percentile approach by applying a framework based on probability of encounter with a group of a given size (see Method section 7). Here we illustrate that both sexes are shifting from small groups at lower density to larger groups at higher densities. For females this involved an increase in groups >13 individual and decrease in groups <5 individuals (the female minimum mean group size). This result is even more striking for males where there was a steep decline in solitary males encountered and a steady increase in groups of males >6 individual in size. This in addition to an increased probability of detecting large groups in females provides support for prediction 3a.

Prediction 3b suggested that increased population density would result in less or no change in dispersion among groups (e.g., [4]). We failed to detect this relationship across observed densities. On the contrary we detected increased dispersion of larger groups of females with increased density (Figure 4). To synthesize the results from the probability of encounter with binned group sizes and the dispersion of groups, this suggested – at least for females – that as density increases groups become larger in size and fewer in number. The group size and dispersion-predation theory (i.e., attack-abatement [48]) predicts a spectrum of response to minimize encounters (avoidance effect) with predators and individual risk of being depredated (dilution effect). Functionally this presents as many small groups or few large groups [48]–[50]. Predation has been shown to affect elk behavior with different effects on group size of males and females [9], [51], [52]. However, these strategies may also be density-specific. During winter (post-mating), male elk are more susceptible to predation [52] and as the encounter probability with predators increases (with population density) males chose to associate with small groups [51] as an anti-predator response [53], rather than be solitary. As such, changes in population density are likely to affect the net benefit of component dilution effects [50], [54], [55]. This is also a plausible driver for the observed negative density dependence in female group size at low population density. Here emergent group properties [56] coupled with predation pressure produce an adaptive minimum group size>1 [47], [57], [58]. At the alternate end of the spectrum, as population size increases it appears that females may mitigate predation risk by forming fewer more dispersed large groups. It seems therefore that female elk in RMNP follow predictions from the group size and dispersion-predation theory as decreased number and increased dispersion of groups is predicted to reduce the probability of encounter with predators [48]. Furthermore, larger groups are predicted to decrease individual probability of being depredated when groups are encountered [48].

Among the costs of social behaviors, the transmission of disease is paramount [12], [18]. Pathogens hitchhike on social contacts between individuals [59]. However, dealing with pathogen transmission in wild population is confounded by complex social behaviors [60], [61]. Proffitt et al. [4] also discussed the importance of social group size for pathogen transmission. In their study, the context is *Brucella abortus* the causative agent of brucellosis. However, for elk in RMNP, the core concern is bovine tuberculosis. Ultimately the fundamental epidemiological models used to understand these diseases have many commonalities. For instance they predict that at a given “critical community size” [62], or population size, pathogens are thought to persist within a population or fade-out (i.e., go extinct), depending on the mode of transmission. For example if transmission is frequency-dependent (i.e., a linear and proportional response to the population size) fade-out may not occur. However, if transmission is density-dependent (i.e., a non-linear response to density) disease may fade-out below a given population size [20]. Population size is strongly correlated to density, especially in bounded populations. At local spatial scales social contacts responsible for pathogen transmission vary with density [26]. Our results suggest that the intra-specific component of pathogen transmission will likely respond to density differently between sexes. Notably, as group size affects the probability of transmission [63] the potential near-linear response to density indicates the lack of a threshold for disease fade-out. Moreover, managers should be cognizant of weak evidence for negative density-dependent response in female group size, which may increase the likelihood of transmission at low population density.

Our study has a number of important caveats. We do not address the implications of group composition or age structure in this article. Composition has been known to affect group size [64]. Rather our focus here is in changes in group size, where determination of group size excluded young-of-the-year. Furthermore, our measure of exposure to group size is insensitive to mixed-sex groups. Excluding the occurrence of yearling males in female groups was, however, valid as observing mixed-sex groups in the area was uncommon (Brook and Vander Wal, personal observation). Given that our measure of exposure to group size is a count of neighboring individuals our estimates of female groups may be biased by at least one individual during the mating season, i.e., breeding male. As the temporal scale of this analysis is annual, we maintain that the bias will be consistent across sampling years (densities). Similarly, inter-season variability is assumed to be consistent across years. Scales of density are inherently complex; we assumed that population density (i.e., size) correlates with local densities of elk. Group size is often an indicator of local density (e.g., [65]). Indeed, here we demonstrate that in some contexts this correlation is not linear. As our analysis is based on total population density (size) as the independent variable we also assumed that sex-ratio of elk remained unchanged throughout the study. Future studies should test whether each sex may respond differently to changes in the density of their own sex more so than changes in total population density was shown here, particularly in species that segregate spatially.

Here we illustrate that sex is a critical factor for understanding non-linear effects population density on group size. In particular this is critical for species known to segregate spatially and sexually [66], [67]. We presented a series of models based on focal individual observations during an intentional population reduction. Our models typically account for more variation in group size than previously realized. Our results also highlight the important implications of population density (competition) for the changing ratio of benefits to constraints of social behaviors, such as grouping. For example, they reinforce the notion that managing disease in wild populations (e.g., bovine tuberculosis as in our study population) may be confounded by social behaviors [26], [60], [61]. Further research that tracks individual fates and quantifies costs and benefits (e.g., through performance measures [68]) to changing group membership will have the potential to unravel critical details related to group living in fission-fusion societies and how group size covaries with population density.

## Supporting Information

Figure S1 **Study area. Riding Mountain National Park** (**RMNP, 3,000 km^2^**) **is located in Manitoba, central Canada.** RMNP is predominantly in the prairie parkland and boreal plains transition zone. Elk (*Cervus canadensis manatobensis*) live primarily within and near the periphery of the preserve. Demarcated within the park are 68 linear transect used to estimate population size.
(TIF)

Table S1 **Supplementary results for mean group size by density analysis.**
(DOCX)

Table S2 **Supplementary results for mean group size by density analysis.**
(DOCX)

Text S1 **Supplementary methods for estimating population size.**
(DOCX)

Text S2 **Supplementary results and discussion for mean group size by density analysis.**
(DOCX)
