# Supplementary figures and images for: Density-Dependent Effects on Group Size Are Sex-Specific in a Gregarious Ungulate
Source: PLoS One. 2013 Jan 9;8(1):e53777. doi: 10.1371/journal.pone.0053777 (PMC3541182; doi:10.1371/journal.pone.0053777)

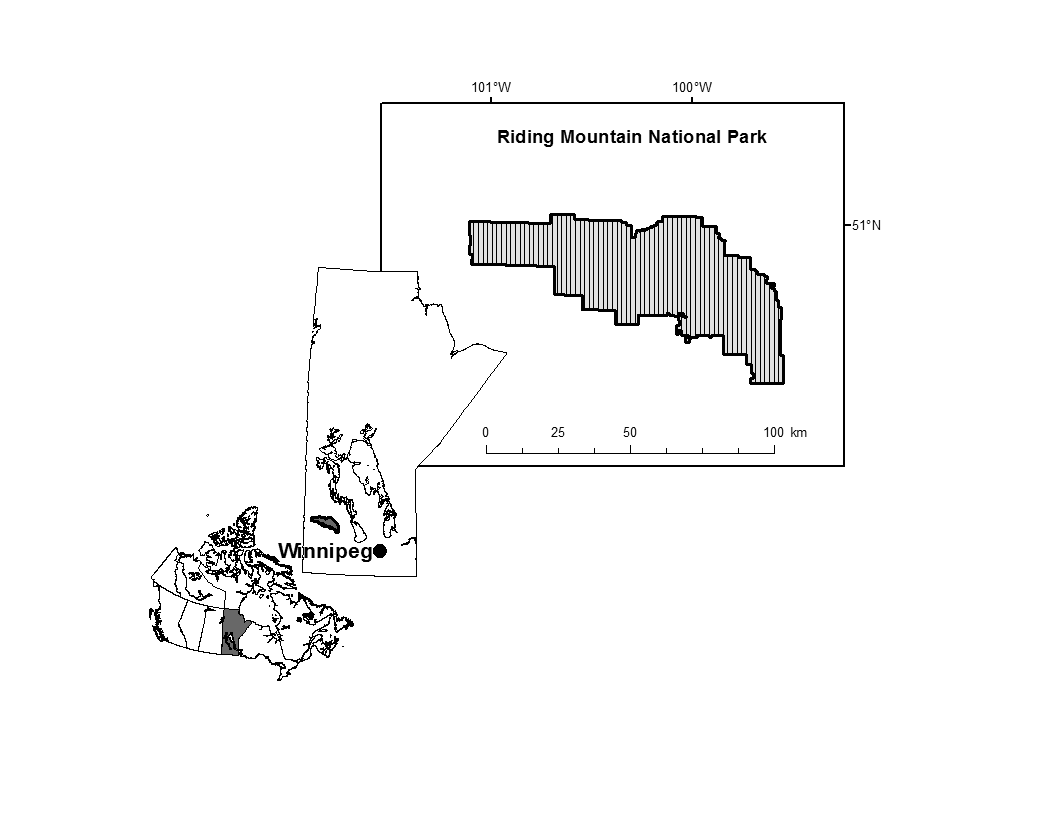

Supplement: Figure S1 — Study area. Riding Mountain National Park (RMNP, 3,000 km2) is located in Manitoba, central Canada. RMNP is predominantly in the prairie parkland and boreal plains transition zone. Elk (Cervus canadensis manatobensis) live primarily within and near the periphery of the preserve. Demarcated within the park are 68 linear transect used to estimate population size. (TIF) [file pone.0053777.s001.tif]
