# Supplementary material for: Density-Dependent Effects on Group Size Are Sex-Specific in a Gregarious Ungulate
Source: PLoS One. 2013 Jan 9;8(1):e53777. doi: 10.1371/journal.pone.0053777 (PMC3541182; doi:10.1371/journal.pone.0053777)
Supplement: Table S1 — Supplementary results for mean group size by density analysis. (DOCX) [file pone.0053777.s002.docx]

**Online Supplementary Material**

(Vander Wal, vanBeest, and Brook: Density-Dependent Effects on Group Size are Sex-Specific in a Gregarious Ungulate)

| **Table S1.** Comparison of frequency – (*FD*) and density – (*DD*) response of mean group size for female (*_F_*) and (*_M_*) elk (*Cervus canadensis*) to population density in Riding Mountain National Park over five years (2003-2004, 2007-2009) during an experimental population reduction. Repeat of regressions from Table 1 without maximum population density included (1.205 elk/km^2^). | | | | | | | |
| --- | --- | --- | --- | --- | --- | --- | --- |
|  | *A priori* Model | | Coefficients and *P-*value | | *R*^2^ | ∆AIC | AIC*_w_* |
| Females | *FD* | $\overline{x}GS_{F}=\beta_{1}(D_{N})+\beta_{0}$ | $\beta_{1}$ =-1.1×10^-4^ | *P* = 0.97 | 0 | 0.37 | 0.63 |
|  | *DD* | $\overline{x}GS_{F}=\beta_{1}(D_{N})+\beta_{2}(D_{N})^{2}+\beta_{0}$ | $\beta_{1}$ = -7.9×10^-2^ | *P* = 0.32 | 0 | 0 | 0.37 |
|  |  |  | $\beta_{2}$ = 1.7×10^-5^ | *P* = 0.32 |  |  |  |
| Males | *FD* | $\overline{x}GS_{M}=\beta_{1}(D_{N})+\beta_{0}$ | $\beta_{1}$ = 1.5×10^-3^ | *P* = 0.12 | 0.47 | 0 | 0.57 |
|  | *DD* | $\overline{x}GS_{M}=\beta_{1}(D_{N})+\beta_{2}(D_{N})^{2}+\beta_{0}$ | $\beta_{1}$ = -2.3×10^-2^ | *P* = 0.38 | 0.52 | 1.13 | 0.42 |
|  |  |  | $\beta_{2}$ = 5.6×10^-6^ | *P* = 0.36 |  |  |  |
