# Supplementary material for: Density-Dependent Effects on Group Size Are Sex-Specific in a Gregarious Ungulate
Source: PLoS One. 2013 Jan 9;8(1):e53777. doi: 10.1371/journal.pone.0053777 (PMC3541182; doi:10.1371/journal.pone.0053777)
Supplement: Text S1 — Supplementary methods for estimating population size. (DOCX) [file pone.0053777.s004.docx]

**Online Supplementary Material**

(Vander Wal, vanBeest, and Brook: Density-Dependent Effects on Group Size are Sex-Specific in a Gregarious Ungulate)

**Supporting Information Text S1**: Supplementary methods for estimating population size.

**Population estimate**: The elk population size was estimated by a 25% cover aerial survey of visible elk. Transects were approximately 200 m wide and conducted annually in January at an altitude of 120 m at 120 km/hr by two trained observers in a fixed-wing aircraft (Figure S1). Standard deviation in population size is estimated by:

$\text{R}=\frac{\text{Y}_{\text{i}}}{\text{Z}_{\text{i}}}\text{ }$[1, estimated density]

$\text{S}z^{2}=\frac{1}{\text{n-1}}\left(\text{Z}^{2}-\frac{\text{Z}^{2}}{\text{n}}\right)\text{ }$ [2, variance between areas sampled]

$\text{S}y^{2}=\frac{1}{\text{n-1}}\left(\text{Y}^{2}-\frac{\text{Y}^{2}}{\text{n}}\right)\text{ }$ [3, variance between animals counted and transects surveyed]

$\text{S}zy=\frac{1}{\text{n-1}}\left(\sum\left(\text{Zi}\times\text{Yi}\right)-\frac{\left(\text{Z}\times\text{Y}\right)}{\text{n}}\right)\text{ }$ [4, covariance between animals counted and transect area]

$\text{s}=\sqrt{\frac{N\left(N-n\right)}{n}\left(Sy^{2}-2RSzy+R^{2}\times Sz^{2}\right)}\text{ }$ [5, standard deviation of density estimate]

Where *N* is the number of sample units in the population (269 transects) and *n* is the number of transects sampled (68); *Z* is the size of the area sampled (745 km^2^), and *z* the size of any one transect (from 8.5 – 24.0 km^2^); *Y_i_* is the number of animals sampled in transect *z_i_*. and *R* the ratio of animals counted to area searched. For further information see [1,2].

During the study period identical transects were flown annually using the same pilot and observers to ensure a consistent estimate of population size across years. We assumed there was little variance in precision of population estimates and as such differences between years remain biologically relevant. As would be expected, we did observe some variation in accuracy according to the calculations above, estimate (SD), within years; 2002: 3617 (365); 2003: 2785 (233); 2004: 2286 (203); 2007: 2016 (213); 2008: 2012 (230); 2009: 2304 (314).

**References**

1. Trottier GC (1987) Riding Mountain Large Mammal Systems Study - Final Report. Winnipeg Manitoba, Canada: Parks Canada. 30 p.

2. Richards LKM (1997) Elk/Moose Population Dynamics in Riding Mountain National Park [M.Sc.]. Winnipeg Manitoba, Canada: University of Manitoba.
