# Supplementary material for: Density-Dependent Effects on Group Size Are Sex-Specific in a Gregarious Ungulate
Source: PLoS One. 2013 Jan 9;8(1):e53777. doi: 10.1371/journal.pone.0053777 (PMC3541182; doi:10.1371/journal.pone.0053777)
Supplement: Text S2 — Supplementary results and discussion for mean group size by density analysis. (DOCX) [file pone.0053777.s005.docx]

**Online Supplementary Material**

(Vander Wal, vanBeest, and Brook: Density-Dependent Effects on Group Size are Sex-Specific in a Gregarious Ungulate)

**Supporting Information Text S2**: Supplementary results and discussion for mean group size by density analysis.

**Frequency- vs. density-dependent changes in mean group size as a function of population density**

We tested whether the results of prediction 2 (density-vs. frequency dependent changes in group size) and negative density dependence in female group size were robust to removal of maximum (Table S1) and minimum (Table S2) observed population densities. The relationship between density and mean group size was lost with the removal of the high density observation (1.205 elk/km^2^); however, was largely unaffected by the loss of the minimum density observations (0.671 elk/km^2^). In both cases the models failed to distinguish between frequency- vs. density dependence (AIC < 2) (Table S2).There was little change for males with the removal of either the maximum (Table S2) or minimum (Table S2) population densities. We argue for the inclusion of the minimum and maximum population densities as the group size observations are well replicated (*n* = 258 and 758 respectively). Further confidence may be gained by records of group size below 0.67 elk/km^2^; however this density arguably represents a near historic low for the elk population in RMNP ([1] and Parks Canada unpublished data, but see [2]).

**References**

1. Rounds RC (1977) Population fluctuations of wapiti (*Cervus elaphus*) and moose (*Alces alces*) in Riding Mountain Park, Manitoba 1950–1976. Canadian Field Naturalist 91: 130–133.

2. Green HU (1933) The wapiti of the Riding Mountain, Manitoba: an ecological study and commentary. Canadian Field Naturalist 47: 105–174.
